# MRI paraspinous skeletal muscle enhancement: A potential imaging biomarker for assessing clinical liver cirrhosis severity

**DOI:** 10.1371/journal.pone.0308520

**Published:** 2024-08-22

**Authors:** Johannes L. du Pisanie, Venkateswaran Ramakrishnan, Vedang Patel, Clayton Commander, Hyeon Yu

**Affiliations:** 1 Department of Radiology, Interventional Radiology, University of North Carolina, Chapel Hill, North Carolina, United States of America; 2 Department of Radiology, University of North Carolina, Chapel Hill, North Carolina, United States of America; 3 Department of Radiology, Interventional Radiology, Baptist Health, Jacksonville, Florida, United States of America; Ascension Sacred Heart Hospital Pensacola, UNITED STATES OF AMERICA

## Abstract

**Purpose:**

To evaluate for correlation between MRI paraspinous muscle (PSM) enhancement and clinical measures of cirrhosis severity (CMCS) utilizing established imaging biomarkers of sarcopenia as comparison.

**Materials and methods:**

Retrospective evaluation of 224 patients (mean age 59.6± 9.7 years, 135 males and 89 females) with liver cirrhosis who underwent contrast-enhanced MRI between August 2021 and August 2022 was performed. Assessed variables included: body mass index (BMI), varices and ascites present on imaging (VPI and API), albumin, total bilirubin (Tbili), international normalized ratio (INR), creatinine, MELD score, as well as history of paracentesis (PH), spontaneous bacterial peritonitis, and variceal bleed (VBH). These variables were compared to PSM skeletal muscle index (SMI), PSM signal fat fractions (sFF), and PSM contrast enhancement fraction (CEFR) calculated on arterial (CEFR-ART), portal venous (CEFR-PV), and delayed (CEFR-DEL) phases collected on MRI.

**Results:**

Patients with MELD>17, PH, and VPI had lower PSM CEFR-ART (0.06vs. 0.11, p = 0.01; 0.07vs. 0.11, p = 0.01; and 0.09vs. 0.13, p = 0.03, respectively). PSM CEFR-ART correlated negatively with MELD. Patients with MELD>17 and PH had lower PSM CEFR-PV (0.16vs. 0.23, p = 0.02; 0.18 vs. 0.23, p = 0.01, respectively). PSM CEFR-PV correlated positively with albumin and negatively with Tbili, INR, and MELD. PSM CEFR-DEL correlated negatively with Tbili and MELD. Patients with API, PH, and VBH had lower PSM SMI (4.68vs. 5.59, p<0.001; 4.37vs. 5.48, p<0.001; 4.78vs. 5.35, p = 0.04, respectively). PSM SMI correlated negatively with Tbili and positively with BMI. PSM sFF correlated positively with BMI, PSM CEFR-PV, and PSM CEFR-DEL.

**Conclusion:**

PSM CEFR is significantly reduced on MRI in patients with clinical manifestations of severe liver cirrhosis. Further investigation into PSM CEFR’s usefulness as an imaging biomarker for evaluating liver disease severity is warranted.

## Introduction

Cirrhosis is a complex, multisystem disease that results in a number of nutritional, metabolic, and physiologic changes [1]. The musculoskeletal system is commonly affected in cirrhosis often manifesting as sarcopenia. Approximately 40–70% of patients with cirrhosis have sarcopenia, which is defined as “a progressive and generalized skeletal muscle disorder associated with an increased likelihood of adverse outcomes including falls, fractures, disability, and mortality” [1–3]. Along with clinical assessment, imaging plays a pivotal role in the diagnosis of sarcopenia which can be characterized in two ways—muscle quantity and quality [2]. The skeletal muscle index (SMI) and skeletal muscle fat fraction (SMFF) have been utilized as imaging biomarkers to measure muscle quantity and quality respectively both of which are associated with poor health outcomes in patients with cirrhosis [4, 5]. Studies have shown that lower liver gadoxetate-contrast enhancement on MRI is a predictor of morbidity and mortality in patients with cirrhosis [6]. A significant difference in SMI calculated using non-contrast versus contrast-enhanced computed tomography has also been shown [7]. Contrast-enhanced ultrasound has also previously been utilized to assess the microcirculation of skeletal muscle [8]. Investigation into MRI skeletal muscle enhancement and its potential association with cirrhosis severity or sarcopenia has not been previously described. SMI and SMFF are frequently measured within the psoas muscle at the L3 vertebra level [3, 9, 10]. Contrast enhanced abdominal MRI is performed in cirrhotic patients to assist in screening for or diagnosing hepatocellular carcinoma (HCC) [11, 12]. The field of view of a standard liver or abdominal MRI often excludes portions of the psoas region; as such, studies have quantified sarcopenia using paraspinous musculature in the upper abdomen [13–15]. Sarcopenia quantification of the paraspinous muscles (PSM) performed at the level of the L1 vertebra or superior mesenteric artery take-off has shown good correlation to quantification performed at the psoas level both on CT and MRI in various patient populations including cirrhotic patients [13–19].

There are many proposed pathophysiological pathways contributing to sarcopenia development in cirrhosis; these include altered protein metabolism, hyperammonemia, hypotestosteronemia, decreased activity, and increased systemic inflammatory cytokines [1]. Alterations in tissue contrast enhancement can be seen in various pathological conditions, from malignancy to inflammation [20, 21]. In a mouse model, it has been shown that injection of a proinflammatory agent into the musculature results in increased contrast enhancement on MRI as compared to a saline control [20]. Higher serum concentrations of certain circulating inflammatory cytokines have also been shown to correlate with clinical cirrhosis severity [22–25]. The cirrhosis-associated immune dysfunction (CAID) model proposes that systemic circulatory dysfunction due to portal hypertension leads to dysregulation of the gut-liver axis, systemic inflammation, and finally, tissue injury with one of the manifestations being sarcopenia [22, 23]. Portosystemic hemodynamic changes have also been seen in cirrhosis. Patients with cirrhosis have been shown to have up to a 300% increase in superior mesenteric artery flow and a 40% decrease in renal blood flow as compared to healthy controls [26]. Given the hemodynamic and immune system dysfunction seen in cirrhosis, we hypothesized that these cirrhotic pathophysiological pathways may also alter skeletal muscle contrast enhancement. As such, we aim to assess for a potential relationship between PSM enhancement and commonly assessed clinical measures of cirrhosis severity (CMCS) utilizing established imaging biomarkers of sarcopenia, SMI and signal fat fraction (sFF), as comparison.

## Materials and methods

### Study population selection

**Fig 1:**
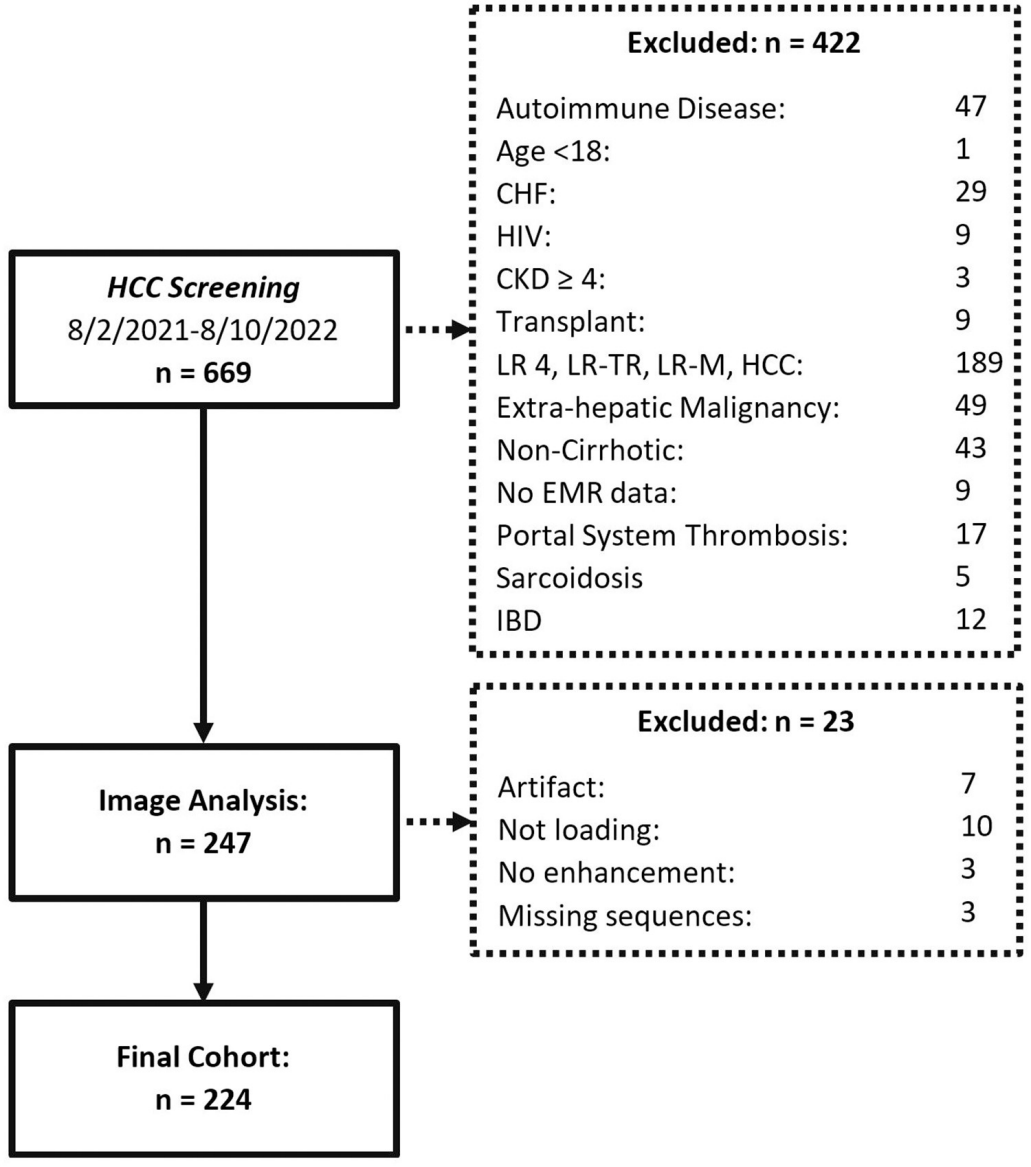
Patient selection flow diagram. Flow diagram depicting the exclusion of patients from the initial sequential cohort. CHF = Congestive heart failure, HIV = human immunodeficiency virus, CKD ≥ 4 = chronic kidney disease greater or equal to stage 4, transplant = patients who underwent any organ transplant, LR 4 = Liver Imaging Reporting and Data System LIRADS (LR)-4 (LR-4), LR-TR = LR treatment response, LRM = LR- likely malignant, and HCC/LR-5 lesions IBD = inflammatory bowel disease. Autoimmune disease was a category of a wide variety of autoimmune diseases including autoimmune hepatitis.

Prior to performing the study, institutional review board (IRB) and Office of Human Research Ethics approval was obtained on August 9 2022. A retrospective review of 669 sequential patients who underwent contrast-enhanced abdominal MRI for hepatocellular (HCC) carcinoma screening between August 2, 2021, and August 10, 2022, was performed. The search term cirrhosis/ HCC screening were utilized to retrieve and populate the patient list for analysis from our institution’s Picture Archiving and Communication System (PACS). An electronic medical record (EMR) review was performed on each of the consecutive patients. Authors had access to information that could identify individual participants during or after data collection via the EMR; Health Insurance Portability and Accountability Act (HIPPA) compliance was maintained throughout data collection, storage, and analysis. Data collection was performed by two 5^th^ post graduate year radiology residents and a 4^th^ year medical student. Data collection was only performed once by any of the three data collectors. Data were first accessed for collection on August 9 2022. Patients were excluded from the study if any of the following criteria were met. These criteria were based on the theoretical potential of these conditions altering paraspinous muscular enhancement through hemodynamic, metabolic, and or systemic inflammatory mechanisms outside of cirrhosis: congestive heart failure (n = 29), human immunodeficiency virus (HIV) infection (n = 9), chronic kidney disease (CKD) stage 4 or greater (n = 3), history of transplant (liver n = 8, renal n = 1), extrahepatic malignancy (n = 49), hepatic malignancy including Liver Imaging Reporting and Data System LIRADS (LR)-4 (LR-4), LR- treatment response (LR-TR), LR- likely malignant (LR-M), and HCC/LR-5 lesions (n = 189), portal system thrombosis (n = 17), autoimmune disease (n = 47), sarcoidosis (n = 5), inflammatory bowel disease (IBD) (Crohn disease n = 2, ulcerative colitis n = 10). Patients were also excluded due to lack of EMR data (n = 9), no history of cirrhosis (n = 43), and age <18 (n = 1). After chart review, 247 patients were included, and imaging analysis was performed (Fig 1). After imaging review, a further 23 patients were excluded due to significant artifact on imaging (n = 7), image loading errors (n = 10), non-enhancement (n = 3), and missing sequences (n = 3) for a total of 224 patients included in the final analysis (Fig 1). Other historical data was then obtained from the EMR, including biological sex, age, race/ethnicity, history of diabetes, thyroid disease history, history of transvenous intrahepatic portosystemic shunt (TIPS), body mass index (BMI), height, cirrhosis etiology, and hepatitis C virus (HCV) infection treatment /sustained viral response (SVR) history. The CMCS data collected from the EMR included patient paracentesis history (PH), history of spontaneous bacterial peritonitis (SBP), and history of variceal bleed (VBH). Imaging CMCS data collected from the MRI report included: the presence and severity of ascites (API) as well as the presence and severity of varices (VPI). If this information was not included in the imaging report, the imaging was manually reviewed by a fifth-year post-graduate year radiology resident, and the API and or VPI was determined. Laboratory CMCS data collected from the EMR included; albumin, international normalized ratio (INR), creatinine, and total bilirubin (Tbili) values closest to the imaging dates. The Model for End-Stage Liver Disease (MELD) score was calculated using the standard equation and the collected laboratory values closest to the image date [27].

### MRI protocol, segmentation, and enhancement assessment

Scans were performed on a Siemens MAGNETOM (Siemens Healthcare, Erlangen, Germany) 1.5 Tesla MRI machine with a body transmit coil. Imaging was processed using Syngo MR E11 (Siemens Healthcare, Erlangen, Germany). T1 In- and Out-of-phase Dixon Sequences were performed with a flip angle of 70°, a 6-millimeter (mm) slice thickness, a 320 x182 acquisition matrix, and a 445 Hertz/pixel bandwidth. The in-phase images were performed with a repetition time of 120 milliseconds (ms) and an echo time of 4.86 ms. The out-of-phase images were performed with a repetition time of 120 (ms) and an echo time of 2.38 ms. Non-contrast and contrast-enhanced images were performed using a T1 VIBE (volumetric interpolated breath-hold examination) fat-saturated sequence with a flip angle of 10°, a 3.5-millimeter (mm) slice thickness, a 320 x144 acquisition matrix, and a 400 Hertz/pixel bandwidth. The contrast agent utilized was gadobenate dimeglumine (Multihance, Bracco Diagnostics Inc. NJ, USA) administered at approximately 0.1 mmol/kg. Care bolus protocol was utilized for contrast injection. This included, Sagittal T1 dynamic contrasted enhanced images obtained at TR 1000 ms and TE 1.37 ms. Upon visualization of the contrast bolus within the descending abdominal aorta by the technologist, T1 fat sat post con arterial phase images were obtained; portal venous timed images were then obtained at approximately 60 seconds and delayed phase images are obtained at approximately 120 seconds.

**Fig 2:**
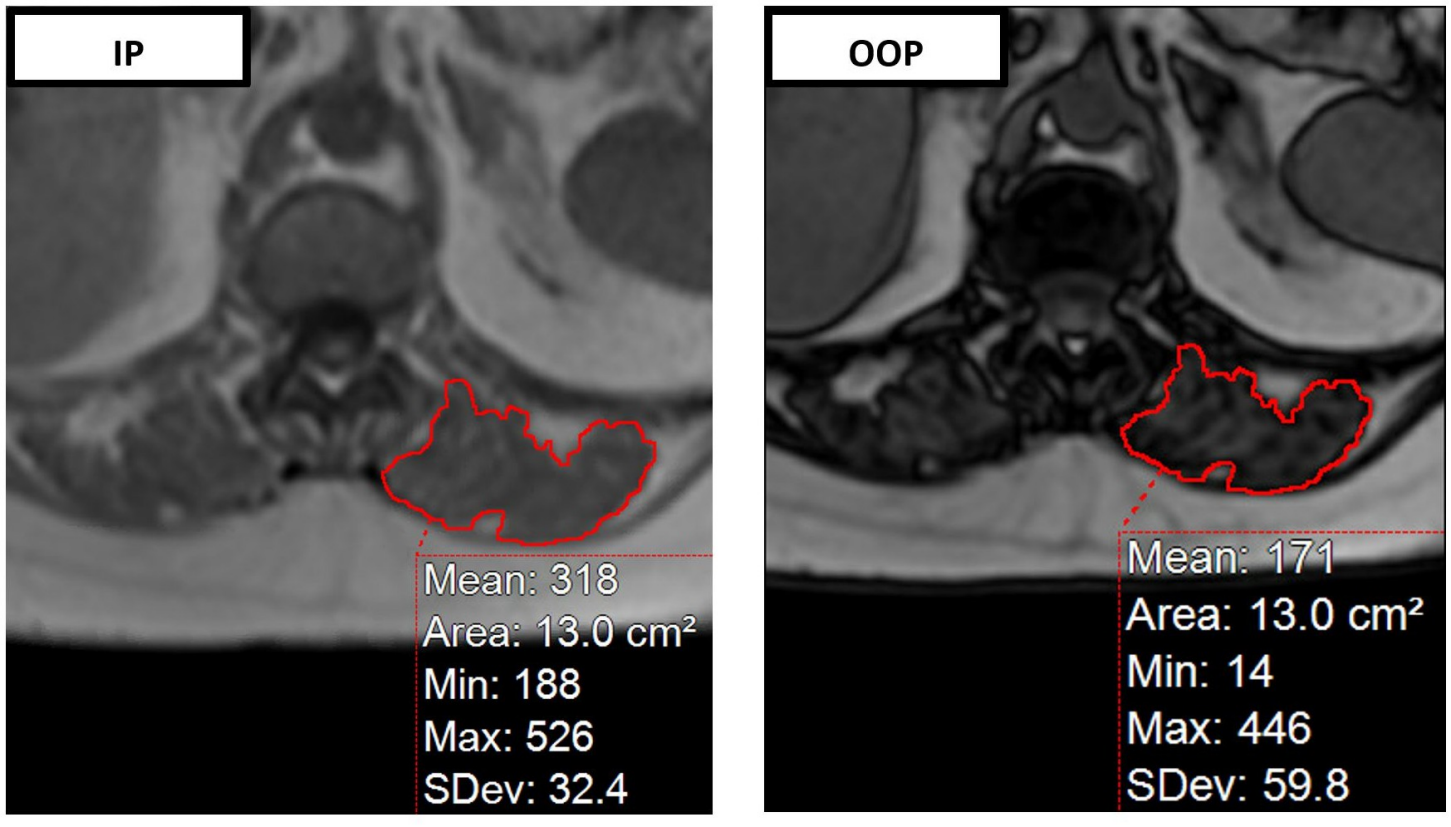
Segmentation of paraspinous muscles to calculate SMI and sFF. Segmentation of paraspinous musculature on in-phase (IP) (left) and out of phase (OOP) (right) paired MRI gradient echo (GRE) sequences at the level of the superior mesenteric artery origin.

Manual segmentation of the bilateral paraspinal muscles (PSM) at the level of the superior mesenteric artery (SMA) origin was performed on both in-phase and out-of-phase MRI sequences utilizing image postprocessing software (Aquarius iNtuition Ver 4.4.13, TeraRecon, Foster City, CA) (Fig 2). The area of bilateral PSMs was averaged and then divided by the patient’s height squared to calculate the PSM skeletal muscle index (SMI; cm^2^m^-2^). The PSM signal fat fraction (sFF) was calculated using the averaged signal intensity (SI) of the bilateral segmented PSMs and the following formula:

$$PSM\;sFF=\;\frac{\left(SI\;in\;phase\right)-\left(SI\;out\;of\;phase\right)}{2*\left(SI\;inphase\right)}$$

Fifty patients were randomly selected from the original data sheet that was filled in by the original 3 reviewers. An attending radiologist separately segmented these patients utilizing the above methods and the muscle area was measured. The calculated concordance correlation coefficient between the two observers (attending radiologist and the data sheet) was 0.994 which shows high correlation.

**Fig 3:**
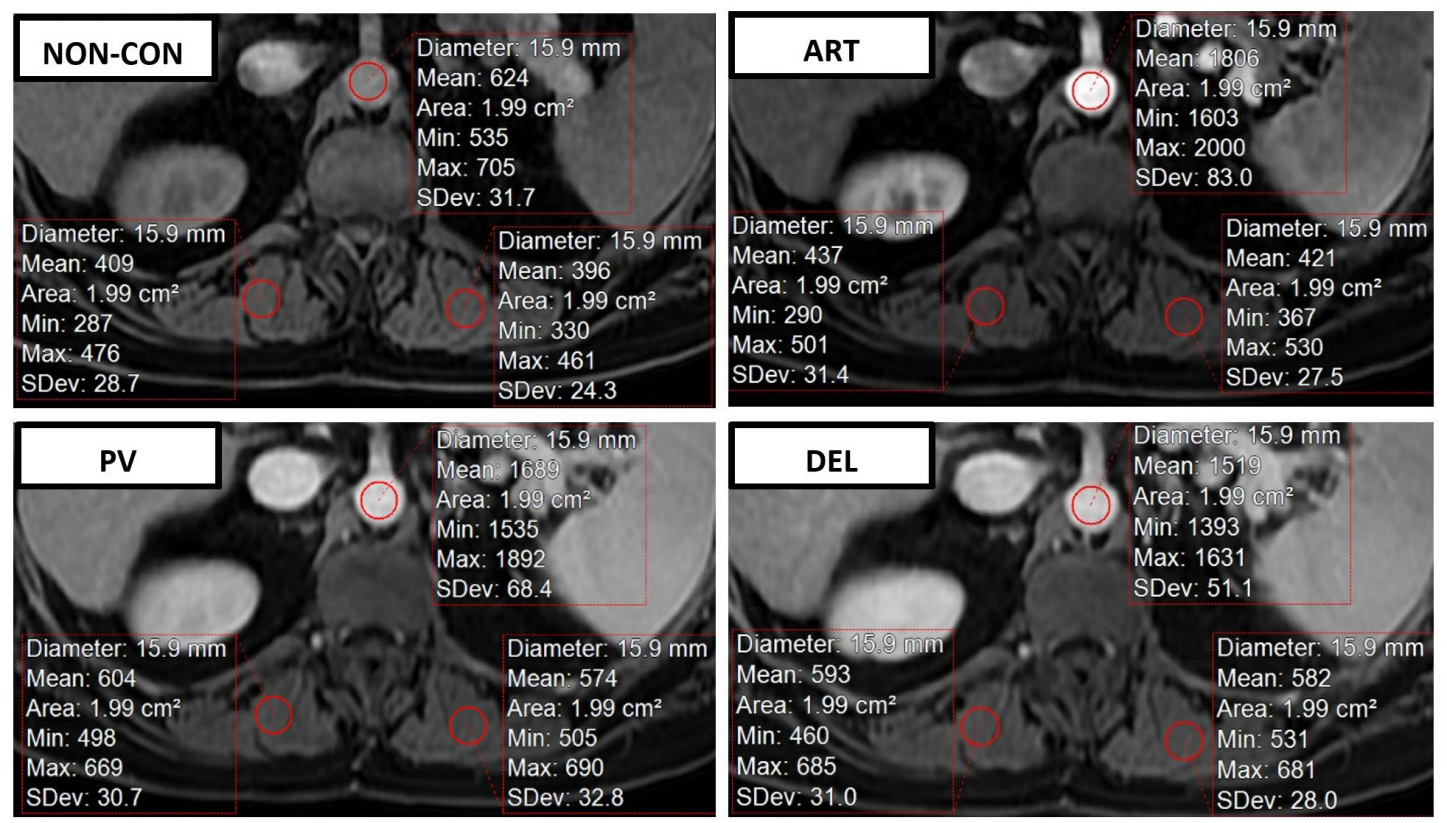
Regions of interest placement to calculate CEFR. Region of interest placement within the bilateral paraspinous muscles and central aorta at the level of the superior mesenteric artery origin for calculation of paraspinous skeletal muscle contrast enhancement fraction (PSM CEFR). Measurements were obtained on non-contrast (NON-CON) (top left), arterial (ART) (top right), portal venous (PV) (bottom left), and delayed (DEL) (bottom right) phases.

Manual regions of interest (ROI) were placed in the bilateral PSMs and central aorta at the level of the SMA origin. This was performed on non-contrast, arterial (ART), portal venous (PV), and delayed (DEL) phases of the contrast-enhanced T1 fat-saturated MRI sequences to assess average PSM enhancement SI utilizing the same image postprocessing software. ROIs utilized had an average area of 1.97 ± 0.02 cm^2^. Care was taken to avoid intramuscular fat, imaging artifacts, and to keep the ROI within the same approximate location on each sequence (Fig 3). Contrast enhancement fraction (CEFR) was calculated for each of the post-contrast T1 fat suppressed sequences (ART, PV, DEL) and utilized for analysis using the following general formula:

$$PSM\;CEFR=\;\frac{\left(SI\;contrast\;enhanced\right)-\left(SI\;noncontrast\right)}{SI\;noncontrast}$$

### Statistical methods

All continuous variables were presented as mean ± standard deviation with range. Categorical variables were presented as absolute numbers and percentages. For parametric data, student t and Pearson’s Chi-squared tests were used. For nonparametric data, Mann-Whitney U and Fisher exact tests were used to compare groups based on independent variables of liver disease severity. Student t and Mann-Whitney U tests were used to compare continuous variables. Chi-squared and Fisher-exact tests were used to compare categorical variables. Shapiro Wilk test was used to determine the distributions of the data. The Pearson correlation test was used to analyze the linear correlation between PSM CEFR, PSM SMI, and PSM sFF. Receiver operating characteristic (ROC) analysis was performed to evaluate the accuracy of the PSM CEFR on each phase, including PSM CEFR-ART, PSM CEFR-PV, and PSM CEFR-DEL in predicting the high MELD score group. Statistical analysis was performed using R (version 4.2.2, R Core Team, 2022, R Foundation for Statistical Computing, Vienna, Austria). The data frame was generated and organized before statistical analysis using the Pandas library in the Python programming language [28, 29]. A p-value of less than 0.05 was considered to indicate a statistically significant difference.

## Results

The patients had a mean age of 60 ± 10 years; 135 were male, and 89 were female. The average MELD score was 10.9 ± 5.3, Albumin was 3.55 ± 0.65 g/dL, Creatinine was 0.93 ± 0.38 mg/dL, Tbili was 1.64 ± 2.11 mg/dL, INR was 1.25 ± 0.35, and BMI was 31 ± 7.3 kg/m^2^ (Table 1).

**Table 1:** Population characteristics.

Population Characteristic	Value
Cohort Size	224
Age (years)	60 ± 10
Sex Assigned at Birth (Male:Female)	135:89
BMI (kg/m^2^)	31 ± 7.3
MELD score	10.9 ± 5.3
Albumin (g/dL)	3.55 ± 0.65
Creatinine (mg/dL)	0.93 ± 0.38
Total Bilirubin (mg/dL)	1.64 ± 2.11
INR	1.25 ± 0.35
Cirrhosis Etiology	
VIRAL	69 (30.8%)
NASH	66 (29.5%)
ETOH	61 (27.2%)
OTHER	11 (4.9%)
ETOH + VIRAL	11 (4.9%)
ETOH + NASH	3 (1.3%)
NASH + VIRAL	2 (0.9%)
VIRAL + OTHER	1 (0.5%)
TIPS	15 (6.7%)
SBP	18 (8%)
Variceal Size	
No	60 (27%)
Small/Mild	128 (57%)
Moderate	18 (8%)
Large	18 (8%)
Variceal Bleed	38 (17%)
Thyroid Dysfunction	19 (8%)
Diabetes	103 (46%)
Ascites Volume	
No	141 (64%)
Trace	32 (14%)
Small/Mild	23 (10%)
Moderate	19 (8%)
Large	9 (4%)
Paracentesis History	47 (21%)

BMI = body mass index; NASH = nonalcoholic steatohepatitis associated cirrhosis; VIRAL = both hepatitis C and Hepatitis B virus infection associated cirrhosis; ETOH = alcoholic associated cirrhosis; OTHER = other etiologies of cirrhosis including cryptogenic; TIPS = transjugular intrahepatic portosystemic shunt; SBP = spontaneous bacterial peritonitis; INR = international Normalized ratio; BMI = Body mass index, MELD = Model for End-Stage Liver Disease. Ascites volumes and variceal sizes are based on MRI reports and interpretation in the event MRI dictation does not specify.

PSM CEFR-ART was lower in patients with MELD > 17 (0.11± 0.13 vs. 0.06± 0.08 p = 0.014), PH (0.07 ± 0.09 vs. 0.11 ± 0.13, p = 0.013), and VPI (0.09 ± 0.12 vs. 0.13 ± 0.12, p = 0.03). PSM CERF-PV was lower in patients with MELD > 17 (PV: 0.23 ± 0.14 vs. 0.16 ± 0.13, p = 0.015) and PH (0.18 ± 0.11 vs. 0.23 ± 0.15, p = 0.008). PSM SMI was lower in patients with ascites (4.68 ± 1.49 vs. 5.59 ± 2.13, p<0.001), PH (4.37 ± 1.62 vs. 5.48 ± 1.99, p<0.001), patients with female sex (5.58 ± 2.11 vs. 4.75 ± 1.61, p = <0.001) and VBH (4.78 ± 1.35 vs 5.35 ± 2.06 p = 0.04). PSM sFF was higher in patients with female sex (0.12 ± 0.08 vs. 0.14± 0.07 p = 0.004) and lower in VBH (0.1 ± 0.06 vs. 0.13 ± 0.08, p = 0.01) (Table 2).

**Table 2:** Univariate analysis of Imaging biomarkers and their association with clinical measures of cirrhosis severity.

		PSM sFF		PSM SMI		PSM CEFR-ART		PSM CEFR-PV		PSM CEFR-DEL	
		$\bar{\text{x}}\pm \sigma$	p	$\bar{\text{x}}\pm \sigma$	p	$\bar{\text{x}}\pm \sigma$	p	$\bar{\text{x}}\pm \sigma$	p	$\bar{\text{x}}\pm \sigma$	p
**Sex**	**Male (M)**	0.12 ± 0.08	0.004*	5.58 ± 2.11	<0.001*	0.09 ± 0.13	0.11	0.21 ± 0.15	0.12	0.24 ± 0.18	0.1
	**Female (F)**	0.14 ± 0.07		4.75 ± 1.61		0.12 ± 0.11		0.24 ± 0.13		0.27 ± 0.13	
**API**	**Yes**	0.12 ± 0.07	0.06	4.68 ± 1.49	<0.001*	0.09 ± 0.12	0.17	0.2 ± 0.15	0.14	0.24 ± 0.2	0.59
	**No**	0.13 ± 0.08		5.59 ± 2.13		0.11 ± 0.12		0.23 ± 0.14		0.26 ± 0.14	
**PH**	**Yes**	0.11 ± 0.07	0.18	4.37 ± 1.62	<0.001*	0.07 ± 0.09	0.01*	0.18 ± 0.11	0.01*	0.23 ± 0.2	0.31
	**No**	0.13 ± 0.08		5.48 ± 1.99		0.11 ± 0.13		0.23 ± 0.15		0.26 ± 0.15	
**VPI**	**Yes**	0.12 ± 0.07	0.06	5.2 ± 1.77	0.57	0.09 ± 0.12	0.03*	0.21 ± 0.15	0.08	0.24 ± 0.17	0.19
	**No**	0.14 ± 0.08		5.39 ± 2.41		0.13 ± 0.12		0.25 ± 0.14		0.27 ± 0.12	
**VBH**	**Yes**	0.1 ± 0.06	0.01*	4.78 ± 1.35	0.04*	0.1 ± 0.15	0.79	0.22 ± 0.18	0.98	0.27 ± 0.25	0.45
	**No**	0.13 ± 0.08		5.35 ± 2.06		0.1 ± 0.13		0.22 ± 0.14		0.24 ± 0.17	
**MELD Score**	**>17**	0.12 ± 0.08	0.59	5.14 ± 2.29	0.79	0.06 ± 0.08	0.01*	0.16 ± 0.13	0.02*	0.19 ± 0.18	0.05
	**≤17**	0.13 ± 0.08		5.27 ± 1.92		0.11 ± 0.13		0.23 ± 0.14		0.26 ± 0.15	

PSM = paraspinous muscle, sFF = muscle signal fat fraction, SMI = skeletal muscle index (cm2/m2), CEFR = Contrast Enhancement Fraction, ART = arterial phase, PV = Portal venous phase, DEL = Delayed phase, API = ascites present on imaging, PH = paracentesis history, VPI = varices present on imaging, VBH = variceal bleed history, MELD = Model for End-Stage Liver Disease, $\bar{\text{x}}=\text{mean}$, σ = standard deviation.

* Statistically Significant result p<0.05.

PSM CEFR-ART negatively correlated with MELD score (R (95%-CI) = -0.15 (-0.28 –-0.02) p = 0.02). PSM CERF-PV correlated positively with albumin (R (95%-CI) = 0.15 (0.02–0.27) p = 0.03), and negatively with Tbili, INR, and MELD score (R (95%-CI) = -0.22 (-0.35 –-0.1) p = 0.001, -0.15 (-0.27 –-0.02) p = 0.02, and -0.21 (-0.33 –-0.08) p = 0.001). PSM CERF-DEL correlated negatively with Tbili and MELD score (R (95%-CI) = -0.2 (-0.32 –-0.07) p = 0.002 and -0.17 (-0.29 –-0.03) p = 0.01). PSM SMI correlated negatively with Tbili and positively with BMI (R (95%-CI) = -0.15 (-0.28–-0.02) p = 0.02 and 0.36 (0.24–0.47) p<0.001). PSM sFF correlated positively with BMI, PSM CERF-PV, and PSM CEFR-DEL (R (95%-CI) = 0.34 (0.22–0.45) p<0.001, 0.13 (0.001–0.26) p = 0.04, and 0.15 (0.02–0.27) p = 0.03) (Table 3).

**Table 3:** Correlations between imaging biomarkers and clinical and laboratory variables.

	PSM sFF		PSM SMI		PSM CEFR-ART		PSM CEFR-PV		PSM CEFR-DEL	
	R (95%-CI)	p	R (95%-CI)	p	R (95%-CI)	p	R (95%-CI)	p	R (95%-CI)	p
**Albumin**	-0.03 (-0.16–0.1)	0.63	0.1 (-0.03–0.23)	0.12	0.06 (-0.07–0.19)	0.38	0.15 (0.02–0.27)	0.03*	0.13 (0–0.26)	0.05
**Creatinine**	-0.05 (-0.18–0.08)	0.45	0.08 (-0.05–0.21)	0.21	-0.06 (-0.19–0.07)	0.36	-0.04 (-0.17–0.1)	0.59	-0.04 (-0.17–0.1)	0.6
**Total Bilirubin**	-0.12 (-0.25–0.01)	0.07	-0.15 (-0.28–-0.02)	0.02*	-0.12 (-0.25–0.01)	0.06	-0.22 (-0.35 –-0.1)	0.001*	-0.2 (-0.32 –-0.07)	0.002*
**INR**	-0.02 (-0.15–0.11)	0.73	-0.01 (-0.14–0.12)	0.89	-0.07 (-0.2–0.06)	0.29	-0.15 (-0.27 –-0.02)	0.02*	-0.1 (-0.23–0.03)	0.12
**MELD Score**	-0.05 (-0.18–0.08)	0.41	-0.06 (-0.19–0.07)	0.39	-0.15 (-0.28 –-0.02)	0.02*	-0.21 (-0.33 –-0.08)	0.001*	-0.17 (-0.29 –-0.03)	0.01*
**BMI**	0.34 (0.22–0.45)	<0.001*	0.36 (0.24–0.47)	<0.001*	0.04 (-0.09–0.17)	0.56	0.02 (-0.11–0.15)	0.72	0.05 (-0.08–0.18)	0.49

PSM = Paraspinous muscle, sFF = signal fat fraction, SMI = skeletal muscle index (cm2/m2), CEFR = Contrast Enhancement Fraction, ART = arterial phase, PV = Portal venous phase, DEL = Delayed phase, Pearson correlation test, R = correlation coefficient, 95%-CI = 95%

confidence interval, p = p value. INR = International Normalized Ratio. MELD = Model for End-Stage Liver Disease, BMI = Body Mass Index.

* Statistically significant result p <0.05.

No statistically significant association was found between the imaging biomarkers and patient age, race, cirrhosis etiology, TIPS presence, thyroid dysfunction, diabetes, or spontaneous bacterial peritonitis history.

## Discussion

PSM CEFR was shown to be significantly decreased on MRI in patients with severe clinical manifestations of liver cirrhosis as compared to those without. PSM SMI and PSM sFF were measured to serve as comparative imaging biomarkers in the assessment of PSM CEFR’s possible relationship with CMCS and sarcopenia. Both PSM SMI and PSM CEFR were shown to be significantly decreased in cirrhotic patients with PH and had a similar significant negative correlation with Tbili. Uniquely, PSM CEFR was also found to be significantly decreased in cirrhotic patients with VPI, and MELD >17 and was shown to have significant positive correlation with albumin and negative correlations with INR, and MELD score; these associations were not seen with PSM SMI or PSM sFF analyses. Unlike PSM CEFR, PSM SMI and PSM sFF were shown to have significant positive correlations with BMI and were shown to be significantly different in cirrhotic patients with VBH as well as in those of the opposite sex. These findings suggest that PSM CEFR may be a useful imaging biomarker for evaluating liver disease severity as measured on contrast enhanced MRI. It is uncertain as to why PSM CEFR is significantly lower in cirrhotic patients with MELD>17, VPI, or PH. Tissue enhancement is a function of blood flow, vascular permeability, and vasculature density; differences seen in PSM CEFR among patients with and without CMCS are likely explained by a derangement in one or more of these processes [30]. The pathophysiologic explanation behind these derangements is likely also multifaceted and possibly driven by altered hemodynamics (i.e. portosystemic shunting, systemic inflammation, and immune dysfunction seen in patients with cirrhosis) [22, 23, 25, 31, 32]. The exclusion criteria utilized were selected to limit inflammatory or hemodynamic effects from patient comorbidities other than cirrhosis which could theoretically alter systemic contrast distribution and enhancement of the PSM. Although the initial thought was that PSM CEFR would be related to PSM SMI directly, no significant correlation was found. PSM sFF only mildly correlated positively with PSM CEFR.

PSM sFF was shown to be significantly higher in female patients with cirrhosis which has been demonstrated in the literature. It is postulated that this is due to differences in intramuscular fat deposition between males and females [4, 33]. PSM SMI was shown to be significantly higher in male cirrhotic patients; this association has also been reported in the literature. A study of cirrhotic patients showed the average male psoas SMI to be 53±0.4 cm^2^m^-2^ and female psoas SMI to be 45±0.7 cm^2^m^-2^ (p = <0.001) [34].

PSM sFF and PSM CEFR showed no significant difference between patients with or without API, whereas PSM-SMI was significantly lower in patients with API. The lack of significant association between myosteatosis and ascites and the significant association between sarcopenia and ascites has also previously been shown in the literature [35–37]. In a study of 473 patients with cirrhosis, similar percentages of those with (47%) and without (42%) myosteatosis on imaging had ascites (p = 0.465) [36]. Another study of 201 cirrhotic patients showed 69% versus 31% of patients with ascites to have sarcopenia versus no sarcopenia respectively (p = <0.0001) [35]. PH was used as a clinical marker of diuretic nonresponsive and or severe ascites, which served to separate these patients from those with less severe ascites. Like in the API analysis, PSM SMI was again shown to be significantly lower in patients with PH; however unlike in the API analysis, PSM CEFR was now also significantly lower in patients with PH. It is postulated that ascites contributes to sarcopenia due to nutritional deficiency due to protein loss, poor oral intake, and early satiety. It has been shown that transjugular intrahepatic portosystemic shunt (TIPS) placement in cirrhotic patients with ascites reverses sarcopenia, which coincides with ascites improvement [1, 38]. This finding gives evidence that PSM CEFR may possibly be more specific of an imaging biomarker for diuretic nonresponsive and or severe ascites.

Both significant and no significant associations between presence of varices in patients with and without sarcopenia and or myosteatosis has previously been shown in the literature [35–37, 39]. Our analysis showed no significant association between varices and PSM SMI and PSM sFF. However, both PSM SMI and PSM sFF were shown to be significantly decreased in patients with VBH. The relationship between VBH and sarcopenia has previously been described in the literature. One study showed a significant increased history of VBH in patients with sarcopenia (p<0.001) [37]. Unlike our analysis, one study showed no significant difference in patients with and without myosteatosis and a history of variceal bleeding (p = 0.26) [33].

A significant association between MELD scores was only noted with the PSM CEFR analysis and not the PSM SMI or PSM sFF analyses. Patients with MELD>17 were shown to have significantly lower PSM CEFR and a significant negative correlation between MELD score and PSM CEFR was seen in all three PSM CEFR groups. MELD score has previously been shown to have no significant association with myosteatosis or sarcopenia in cirrhotic patients [4, 34, 36, 40]. However, some studies have shown a significant association between myosteatosis and sarcopenia with MELD score in cirrhotic patients [33, 35, 37]. It is likely that our analysis may not have shown significant association between PSM SMI and PSM sFF and MELD score as we did not include defined cut-off values for sarcopenia or myosteatosis, as was performed in some of these analyses, but rather analyzed PSM SMI and PSM sFF as continuous variables.

Both PSM SMI and PSM CEFR were shown to have similar significant negative correlation with Tbili. This positive association between sarcopenia and Tbili has been shown previously in the literature [37, 40]. No significant association between myosteatosis and Tbili has also been previously shown in the literature [36]. Significant correlations were seen between PSM CEFR and albumin and INR; positive and negative respectively. No significant association was seen between PSM SMI and PSM sFF when compared to albumin and INR; this is also in keeping with what has been shown in the literature [33, 36, 40]. However, one study did show a significant positive and negative association with sarcopenia and INR and albumin respectively [35]. Another only showed a significant positive association between INR and sarcopenia [37]. Again, this may be related to treating PSM SMI and PSM sFF as continuous variables rather than defining cut-off values for sarcopenia or myosteatosis for these variables. These findings suggest that PSM CEFR may have a close relationship with biochemical/laboratory markers of liver disease severity and nutritional status. BMI was shown to only significantly positively correlate with the PSM SMI and PSM sFF groups which has also been previously shown in the literature [33, 34, 36, 37]. These findings suggests that PSM CEFR may be less influenced by differences in body composition and sex as previously mentioned above.

There are some limitations of this study that must be highlighted. First and foremost, the retrospective design of our investigation weakens the significance of our results and a well-designed prospective control trial with a larger more diverse patient cohort is needed to aid in validation. Although our selection criteria aimed to exclude confounding comorbidities which could alter PSM contrast enhancement other than cirrhosis, there may have been some unaccounted-for factors that could contribute to this bias and confounding. The observational nature of our investigation also limits the ability of our results to assess the causality behind the above-described findings. The smaller number of female patients as compared to male patients may also limit the significance of the results when comparing differences in imaging biomarkers and sex as well as the generalizability of our results to a broader more diverse population. Finally, the lack of a non-cirrhotic-control group to compare our results to and the exclusion criteria utilized also limit the generalizability of these results.

In conclusion, this investigation has shown there to be a significant difference between PSM CEFR in cirrhotic patients with and without certain CMCS, which has not previously been demonstrated in the literature. PSM CEFR was only found to have similar significant associations with PSM SMI in the PH and Tbili analyses. However, unlike PSM sFF and PSM SMI, PSM CEFR was shown to have a uniquely significant association with the MELD score, Albumin, and INR in our patient cohort. This suggests a relationship between PSM CEFR and biochemical markers of liver cirrhosis severity. Unlike PSM SMI and PSM sFF, PSM CEFR showed no difference when comparing males and females and no correlation with BMI suggesting that PSM CEFR may be less influenced by body composition and or differences due to sex in our patient cohort. Interestingly, there was no significant positive or negative correlation found between PSM CEFR and PSM-SMI; PSM CEFR only correlated mildly positively with PSM sFF when compared directly. The clinical implication of these results as they stand are limited given the lack of prospective or control validation as well as the small cohort; however, these observational results give merit to further investigation into PSM CEFR’s usefulness as an imaging biomarker for assessing cirrhosis severity. Further investigation may give insight into PSM CEFR’s pathophysiology, association with morbidity and mortality, and predictive capabilities in a larger non-selected group of cirrhotic and non-cirrhotic patients.

## Supporting information

S1 Datasheet Data sheet including all variables collected on the final analyzed patient cohort (n = 224). (CSV)
